# Pilot study for generating and assessing nomograms and decision curves analysis to predict clinically significant prostate cancer using only spatially registered multi-parametric MRI

**DOI:** 10.3389/fonc.2023.1066498

**Published:** 2023-01-24

**Authors:** Rulon Mayer, Baris Turkbey, Peter Choyke, Charles B. Simone

**Affiliations:** ^1^ Department of Radiation Oncology, Perelman School of Medicine, University of Pennsylvania, Philadelphia, PA, United States; ^2^ OncoScore, Garrett Park, MD, United States; ^3^ Molecular Imaging Branch, National Institutes of Health (NIH), Bethesda, MD, United States; ^4^ Department of Radiation Oncology, New York Proton Center, New York, NY, United States

**Keywords:** prostate cancer, multi-parametric magnetic resonance imaging (MP-MRI), Gleason score (GS), signal-to-clutter ratio (SCR), regularization, nomograms, decision curve analysis, multiple variable regression

## Abstract

**Background:**

Current prostate cancer evaluation can be inaccurate and burdensome. To help non-invasive prostate tumor assessment, recent algorithms applied to spatially registered multi-parametric (SRMP) MRI extracted novel clinically relevant metrics, namely the tumor’s eccentricity (shape), signal-to-clutter ratio (SCR), and volume.

**Purpose:**

Conduct a pilot study to predict the risk of developing clinically significant prostate cancer using nomograms and employing Decision Curves Analysis (DCA) from the SRMP MRI-based features to help clinicians non-invasively manage prostate cancer.

**Methods:**

This study retrospectively analyzed 25 prostate cancer patients. MP-MRI (T1, T2, diffusion, dynamic contrast-enhanced) were resized, translated, and stitched to form SRMP MRI. Target detection algorithm [adaptive cosine estimator (ACE)] applied to SRMP MRI determines tumor’s eccentricity, noise reduced SCR (by regularizing or eliminating principal components (PC) from the covariance matrix), and volume. Pathology assessed wholemount prostatectomy for Gleason score (GS). Tumors with GS >=4+3 (<=3+4) were judged as “Clinically Significant” (“Insignificant”). Logistic regression combined eccentricity, SCR, volume to generate probability distribution. Nomograms, DCA used all patients plus training (13 patients) and test (12 patients) sets. Area Under the Curves for (AUC) for Receiver Operator Curves (ROC) and p-values evaluated the performance.

**Results:**

Combining eccentricity (0.45 ACE threshold), SCR (3, 4 PCs), SCR (regularized, modified regularization) with tumor volume (0.65 ACE threshold) improved AUC (>0.70) for ROC curves and p-values (<0.05) for logistic fit. DCA showed greater net benefit from model fit than univariate analysis, treating “all,” or “none.” Training/test sets achieved comparable AUC but with higher p-values.

**Conclusions:**

Performance of nomograms and DCA based on metrics derived from SRMP-MRI in this pilot study were comparable to those using prostate serum antigen, age, and PI-RADS.

## Introduction

For prostate cancer, deciding to treat clinically significant disease or to monitor benign lesions or low risk invasive disease (1) requires correct assessment in order to properly manage the disease. A large number of factors, such as Gleason score, prostate serum antigen (PSA) (2–4), metadata (5) such as patient age, family history, tumor size (6), clinical stage and visual inspection of images of the lesion (7–11), etc. contribute to a patient’s evaluation, but they vary in their correlation to disease status. The large number and variation of contributing factors among patients can complicate cancer management and confuse the clinician and patient. A nomogram (12–14) is a graphical depiction that quantitatively combines a number of factors to help summarize a patient’s status and simplify the assessment. The nomogram produces a probability distribution for the likelihood of serious disease that is tailored for each individual patient. Along with a nomogram, a Decision Curve Analysis (DCA) (15) can refine and enhance the management of the patient by providing a graph to suggest when or if to apply certain procedures.

Further complicating patient management, the factors that contribute to patient evaluation can also potentially discomfort the patient and produce side effects (16). Specifically, a prostate biopsy, currently the standard assessment, can cause hemorrhaging, pain, and infection, and it can possibly miss properly sampling the tumor (17). To elevate patient assessment, imaging, such as MRI, can non-invasively display the entire image and tumor with minimal patient discomfort. Specifically, qualitative assessment of multiple modalities of MRI or Multi-Parametric MRI (MP-MRI) employ trained radiologists who follow the Prostate Imaging Reporting and Data System (PI-RADS) protocol (7). Recently, PI-RADS assessments have been incorporated into nomograms and achieved significant accuracy in predicted disease outcomes (18–23). However, the quality of the PI-RADS assessment can vary depending on the training or experience of the radiologist examining a patient’s image (24). A more quantitative, robust approach is desired.

Recently (25–30), algorithms have been applied to spatially registered MP-MRI to assess prostate tumors. These algorithms exploit the vectoral nature of each voxel in the prostate organ, unlike others that process individual modalities. Each voxel is treated as a vector, not a scalar. The recent studies determined the prostate tumor’s Gleason score (25–30), tumor volume (26), eccentricity (shape) (27), and Signal-to-Clutter Ratio (SCR) (29).

This study is the first to use spatially registered MP-MRI as input information for a nomogram and for DCA. This study used patient data from The Cancer Imaging Archive (TCIA) (37, 38) that is composed of twenty-six consecutive patients who had biopsy proven adenocarcinoma of the prostate, had undergone MRI scan, and histological examination of wholemount prostatectomy. For this study, clinically significant (insignificant) prostate cancer was defined by the pathology assessment of Gleason scores >=4+3 (<=3+4). The present retrospective work does not use other clinical data (18–23) such as age, PSA nor use PI-RADS as input for the nomogram. Instead, the nomograms use various combinations of eccentricity, filtered and regularized SCR, and tumor volume indicators to find the probability that the prostate tumor is highly aggressive. This study extends and builds upon earlier work (28, 30) that examined multivariable regression fits to Gleason scores in order to generate a clinical tool to aid in the management of prostate cancer. The nomogram and decision curve analysis were quantitatively assessed by computing the Area Under the Curve (AUC) for the Receiver Operator Characteristic (ROC), p-values.

## Methods

### Overall description

**Figure 1:**
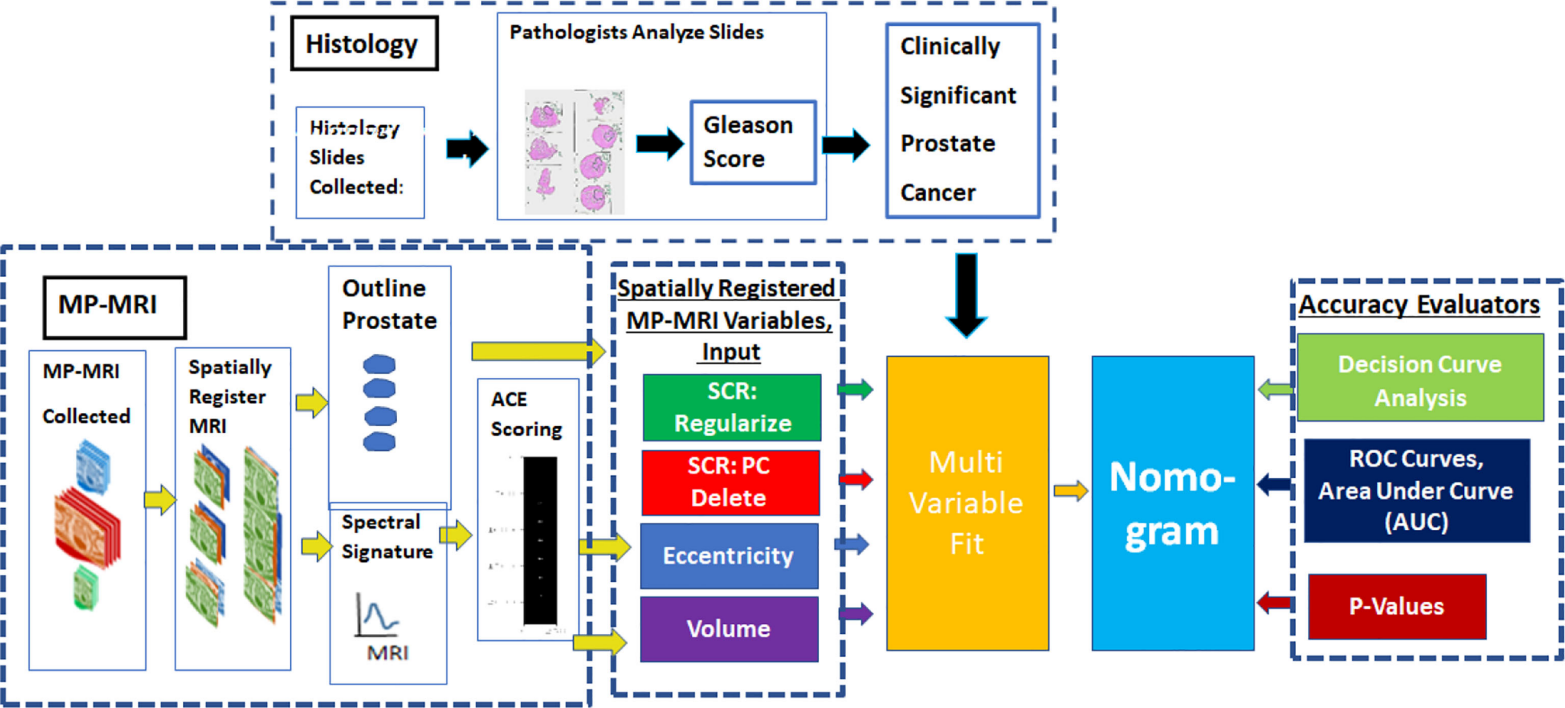
provides an overview of the methodology to generate a nomogram from metrics derived from spatially registered MP-MRI (25–30) along with accompanying performance evaluations. The main components in the summary are described in greater detail below. The independent variable for the multivariable fit originates from spatially registered MP-MRI and the dependent categorical variable Clinically Significant Prostate Cancer derived from Gleason score and pathology exam of histology of the resected prostate.

**Figure 1** provides an overview of the methodology to generate a nomogram from metrics derived from spatially registered MP-MRI (25–30) along with accompanying performance evaluations. The main components in the summary are described in greater detail below. The independent variable for the multivariable fit originates from spatially registered MP-MRI and the dependent categorical variable Clinically Significant Prostate Cancer derived from Gleason score and pathology exam of histology of the resected prostate.

Sequences of MRI (T1, T2, Dynamic Contrast Enhancement, Diffusion) were collected from each patient. The images were rescaled, cropped, translated, and resampled to form spatially registered multispectral cubes. These cubes were then stitched together to form spatially registered hypercubes. From visual inspection, the normal prostate was digitally outlined using an axial view to form the normal tissue or background. A vector tumor signature was taken from certain voxels identified in the colorized registered hypercube (25–30) and inserted into the Adaptive Cosine Estimator, and a threshold (25–30) was applied to find the tumor volume and eccentricity.

The nomogram (text box colored as baby blue in **Figure 1**) receives input from a multi-variable fit (yellow text box). Multivariable regression fits independent variables from spatially registered MP-MRI to the independent variable Gleason score. The independent variables are Regularized SCR (green), SCR with principal component filtering (red), tumor eccentricity (blue), tumor volume (purple) and combined in a variety of permutations to achieve an optimal fit. The dependent variable is categorical Clinically Significant Prostate Cancer, related to the Gleason score and is derived from pathology, not MRI. A pathologist determines the Gleason score from microscopic inspection of histology slides of wholemount prostatectomy.

To assess the multivariable regression fit and the nomogram, Receiver Operator Characteristic curves were generated and the Area Under the Curve (AUC) was computed. The coefficient of determination (R2) between the independent and dependent variables was computed along with the probability for the null hypothesis (p-value). To further assess and extend the clinical application of the nomogram, a Decision Curve Analysis was computed to find the net benefit for applying the nomogram.

The Methods qualitatively describes the individual components of anomaly detector generator and assessment. The Appendix summarizes the mathematics used to generate the components. More details can be found in the cited references.

### Study design and population

Patient data from prostate tumor MRI and histology from whole mount prostatectomy specimens were collected and stored through The Cancer Imaging Archive (TCIA) (37, 38), affiliated with The National Institutes of Health (NIH). This study followed the Declaration of Helsinki (as revised in 2013). This study is compliant with the Health Insurance Portability and Accountability Act. The NIH Institutional Review Board approved this retrospectively designed single institution study and determined that individual consent for this retrospective analysis was not required. Twenty-six consecutive patients in the TCIA database were assessed. All patients had biopsy proven adenocarcinoma of the prostate, with median patient age of 60 years (range, 49 to 75 years), with a median PSA of 5.8 ng/mL (range, 2.3 to 23.7 ng/mL) and with median GS of 7 (range, 6 to 9). Eighteen of the 26 patients had tumor sizes >1 cc. One patient did not uptake the contrast material used for Dynamic Contrast Enhancement. This study did not place restrictions on tumor location within the prostate. Robotic assisted radical prostatectomy was performed following MRI without any intervening treatment. All cases were anonymized for subsequent analysis.

### Whole mount prostatectomy and histology

The whole mount prostatectomy histology has previously been described (39–41). Following radical prostatectomy, the specimen was fixed at room temperature in formalin for 2 to 24 hours, placed in the customized 3D mold, and sliced in sections with a separation of 6 mm. in the axial direction (corresponding to the MRI axial plane section). The individual tumor foci, dimensions, and GSs from the histology slides were independently determined by two experienced pathologists blinded to the MRI results. As in earlier studies (25–30) and to better reflect the patient’s status, a patient’s GS was a weighted average (based on histology blob size) of the GSs assessed by the pathologists.

### Magnetic resonance imaging

The MRI collection was composed of diffusion weighted images (DWIs), dynamic contrast enhanced (DCE), and structural (T1, T2) images. The pulse sequences were described in earlier studies (39–41). Triplanar T2W turbo spin echo, DW MRI, and axial pre-contrast T1-weighted axial 3D fast field echo DCE MRI sequences were part of this MRI protocol. The detailed sequence parameters were described in a prior study (41). The mean interval between MRI and radical prostatectomy was 60 days (range, 3 to 180 days).

### Image processing, pre-analysis

The DCE are a time series of images follow contrast material in tissues over several hundred seconds following injection. A portion of tumors may be identified through analysis of DCE and exploiting the unique tumor physiology. The tracer concentration in the tissue that supplies and empties through the tumor vasculature is described by a simple two compartment model (25, 42, 43). For longer times (>50 seconds) than the time to reach the contrast material peak uptake in a tumor, every voxel was fitted with an exponentially decay function to form the washout (k_ep_).

The MRI images were digitally resized (25–30) to 1 mm resolution in the transverse direction. In the axial direction, the slices were resized to 6 mm spacing and aligned using resampling based on the known location of patient’s table position. Due to the short time interval between scans (<20 minutes), only small rigid adjustments (minor transverse translation) were applied to the structural, diffusion, and DCE images. A “cube” was formed from stacked individual slices that were scaled, translated, resliced and were thereby spatially registered at the voxel level. These “three dimensional” (two transverse directions plus spectral composed of MP-MRI modalities) cubes were then “stitched” together into a narrow three-dimensional hypercube in order to depict the entire MRI scan. The spectral content of the hypercube had 7 components (25–30) [T1 (pre-contrast), T1 (maximum contrast), T2, ADC, DWI-high B (B=1,000 s/mm^2^), Washout or k_ep_ from DCE].

### Eccentricity calculation

Custom software (coded in Python 3) was used to calculate the eccentricity (27, 28) for every labeled blob. The moment of inertia matrix I for the kth blob was computed. From the eigenvalues of the moment of inertia I, the largest eigenvalue was assigned to the large axis l_k_ and the second eigenvalue was assigned to the transverse moment s_k_. The eccentricity E_k_ for the kth blob is a weighted difference of the major axis and minor axis. Eccentricity values E_k_ range from 0 (spherical shape) to 1 (line). For more details see References (27, 28).

### Overall quantitative metrics description: SCR

Instead of relying on trained radiologists to visually inspect multiple MRI images, the Signal to Clutter Ratio quantitatively assesses tumors departure from normal prostate tissue. The SCR formulation combines all components of the MP-MRI. But in addition, the SCR formulation uses the covariance matrix, to correct and account for correlations among the different components (for example, the correlation between ADC and DWI) to get a true measure of the aggregate contribution of each. The Appendix summarizes some of the mathematics behind the SCR algorithm. For more details see (29, 31, 34–36).

### SCR: Filtering noise

Computing the SCR covariance matrix generates principal components (34). Principal component are linear combinations of all MRI components but are orthogonal or totally decorrelated from each other. The principal components are ordered based on their eigenvalue or statistical variation. Well resolved images have eigenvalue and high variation. In contrast, the noisy principal components have small eigenvalues. Noise is reduced by filtering and eliminating the noisy (low eigenvalue) principal components resulting in a more accurate RX calculation. The Appendix summarizes some of the mathematics behind the filtering of principal components. For more details see (29, 31, 35).

### Regularization and shrinkage

Regularization is another way to correct for the imperfections of the computed covariance matrix. The statistics describing the background (normal prostate) should follow a normal distribution. However, the analytic formula for the covariance matrix results in only an approximation. The goal of shrinkage regularization (29, 36) is to perturb the original covariance matrix CM(γ) by mixing in a diagonal matrix with a mixing parameter γ to generate a regularized or modified regularized covariance matrix. The appropriate γ is chosen to maximize the normal distribution. Regularized or modified regularized covariance matrix generation follow the same procedure but differ in the mixing diagonal matrix. The Appendix summarizes some of the mathematics behind regularization. For more details see (29, 36).

### Tumor volume measurements, supervised target detection

The Supervised target detection algorithm or ACE was applied to the spatially registered MRI (26) and was used to determine the tumor volume. Voxels exceeding a threshold for ACE scores are assigned to tumor and normal tissue are assigned to ACE scores residing below the threshold. The number of voxels exceeding the threshold (tumor) were counted and converted to volume based on the MRI spatial resolution The Appendix summarizes some of the mathematics behind tumor volume computation. For more details see (26).

### Logistic regression

A logistic regression fit (44, 45) was applied to the dependent categorial variable CsPCa, using all combinations of the continuous independent variables (eccentricity, SCR, volume). The GS derived from the pathological assessment of histology slides from prostatectomy. The clinically significant PCa (CsPCa) was assigned to Gleason score >=4+3 and the clinically insignificant PCa (CiPCa) is assigned to<=3+4 This study only reports the combination of independent variables that achieved the highest performance in earlier studies (27–30). The eccentricity from the largest blob used ACE threshold 0.45. SCR includes cutoff from three and four principal components, regularized SCR and modified regularization. The volume derived from MP-MRI used ACE threshold 0.65. The coefficient of determination R2 assesses the fit. In addition, the quality of fit was assessed by computing the F-value and affiliated P value (44, 45).

### Receiver operator characteristic

The Receiver Operator Characteristic curve summarizes (46) and helps assess a binary classifier by plotting the probability of target detection (or sensitivity) against the false alarm probability (or 1-specificity) for all threshold settings. The classifier’s accuracy is assessed by comparing the multivariable logistic regression fitted results with the pathologist’s Gleason score determination for each patient.

The ROC vertical axis (Sensitivity) surveys the patients with clinically significant prostate cancer (CsPCa) and determines whether the patient’s prostate cancer status is correctly identified by the logistic regression for a given threshold. The horizontal axis (False Alarm probability or 1-Specificity) displays the relative accuracy for determining the status of patients with clinically identified as insignificant prostate cancer (CiPCa) for a given probability threshold. The ROC curve is monotonically increasing. If feasible, the best ROC curve value would be 100% target detection and 0% False Alarm probability (upper left corner for the ROC curve). The Area Under the Curve (AUC) is used to assess classifier and ranges from 0 (poor performance) to 1 (optimal performance).

### Nomogram and decision curve analysis

A nomogram (12–14) is a two-dimensional calculating device designed to graphically depict a statistical prognostic model that generates a probability of a clinical event. Nomograms use biologic and clinical variables. In this study, the nomograms employ a logistic regression to model the probability that a prostate tumor is clinically significant. Each variable is listed separately, with a corresponding number of points assigned to a given magnitude of the variable. The individual points are summed from each variable to generate the total number of points for all variables. The total point score is projected onto the scale of outcome. Nomograms can be tailored to an individual patient and potentially reduce biopsies and their morbidity. They are widely used for cancer prediction.

Decision Curve Analysis (15) plots the net benefit associated with a model against the model’s threshold probability. Net benefit is a weighted difference combination of True and False identifications of clinically significant prostate cancer, weighted by the threshold probability.

Alternatively, the threshold probability is the minimum probability of an event at which a decision-maker would take a given action, i.e. the probability of cancer at which a doctor would order a SRMP MRI scan. A lower threshold probability means a patient’s greater concern about cancer, while a higher threshold reflects greater concern about a patient’s aversion to SRMP-MRI. A positive classification is defined by whether predicted probability is at least as great as the threshold probability. As a reference (and by convention), the display includes the results of the default strategies of assuming that all or no observations are positive as a function of threshold probability.

Decision Curve Analysis assesses the clinical value of a predictor, unlike other evaluation statistical methods. Applying decision curve analysis can determine whether using a predictor to make clinical decisions like performing a SRMP MRI scan will provide benefit over alternative decision criteria, given a specified threshold probability

## Results

**Table 1** summarizes the assessments of 25 consecutive patients with contrast enhanced MRIs. Patients were assessed for the best fitting combinations of metrics derived from spatially registered MP-MRI to the Risk of PCa categorical variable. The independent variables include tumor eccentricity using an ACE threshold of 0.45 (Ecc), SCR using regularization (Reg), SCR using Modified SCR (Mod_Reg), SCR after filtering out 3 PC (3PC), and tumor volume (Vol) using 0.65 for the ACE threshold. The dependent categorical variable (Risk of PCa) was taken from the pathology determined Gleason Scores. The number of variables identified for each fit. **Tables 1**, **2** lists each fit’s F values and associated probability of null hypothesis p-values, coefficient of determination (R2), Area Under the Curve (AUC) for the Receiver Operator curves and the AUC’s 95% Confidence intervals Lower Level (LL) and Upper Level (UL). The fits have statistical significance (p-values<0.01), achieve high coefficient of determination (R2>0.60), high AUC (>0.85) but large confidence interval (0.20).

**Table 1:** Summary of Logistic Regression fits for All patients.

Independent Variables	# Variables	F Value	p-value	R2	AUC [95% LL, 95% UL]
3PC+Vol	2	16.08	0.0003	0.664	0.912 [0.792, 1.00]
					
Ecc+3PC	2	14.43	0.0007	0.614	0.882 [0.719, 1.00]
					
Ecc+Mod Reg+3PC	3	14.47	0.0023	0.615	0.882 [0.719, 1.00]
					
Ecc+ Reg+3PC	3	14.99	0.0018	0.631	0.882 [0.719, 1.00]
					
Ecc+3PC+Vol	3	16.87	0.0008	0.687	0.919 [0.799,1.00]
					
Ecc+ Reg+3PC+Vol	4	17.03	0.0019	0.691	0.919 [0.799,1.00]
					
Ecc+Mod Reg+3PC+Vol	4	17.02	0.0019	0.691	0.926 [0.804,1.00]

Analyzing all patients, Summary of Best Regression fits of combinations eccentricity, SCR, and Volume to Gleason score. AUC, Area Under Curve; R2, coefficient of determination; LL, Lower Limit Confidence Interval; UL Upper Limit 95% Confidence Interval; ECC, eccentricity(0.45 ACE Threshold); Mod_Reg, Modified Regularization; SCR, Reg, Regularized SCR; Vol, Volume (0.65 ACE threshold).

**Table 2** replicates **Table 1** except using a greater number of independent variables(eccentricity, SCR, volume) and the analysis follows a test set (12 consecutive odd numbered patients) that used the fitted parameters from training 13 consecutive even numbered patient. Like **Table 1**, high AUC scores (>0.85) are achieved. However, p-values were higher, and the coefficients of determination were lower.

**Table 2:** Summary of Logistic Regression fits for Training, Test Sets.

Independent Variables	# Variables	F Value	p-value	R2	AUC (Train)	AUC (Test) [95% LL, 95%UL]
						
3PC+Vol, Train-Test	2	5.63	0.0598	0.496	0.861	0.969 [0.882-1.00]
						
Ecc+ Reg+3PC, Train+Test	3	6.29	0.0984	0.541	0.889	0.906 [0.702-1.00]
						
Ecc+ Reg+3PC+Vol, Train+Test	4	6.98	0.137	0.586	0.944	0.938 [0.791-1.00]

Analysis of Training and Test Cases, Summary of Best Regression fits of combinations of eccentricity, SCR, and Volume to Gleason score. AUC, Area Under Curve; R2, coefficient of determination; LL, Lower Limit Confidence Interval; UL Upper Limit 95% Confidence Interval; ECC, eccentricity(0.45 ACE Threshold); Mod_Reg, Modified Regularization SCR; Reg, Regularized SCR; Vol, Volume (0.65 ACE threshold).

**Figure 2:**
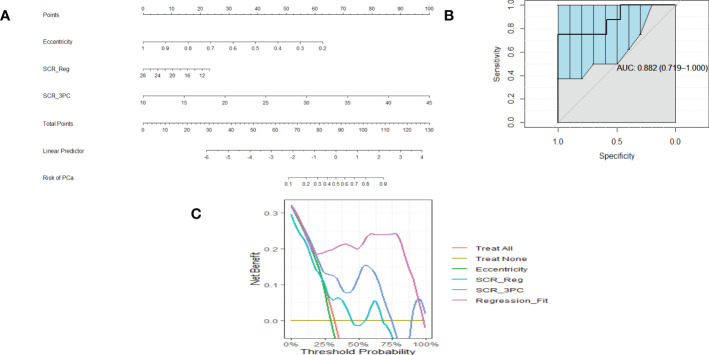
**(A)** is a nomogram resulting from logistic fit to Gleason score with eccentricity, regularized SCR, and SCR with 3 PC removed. **(B)**. ROC curve for fitting eccentricity, regularized SCR and SCR after 3 PC removed, Area Under the Curve (AUC) and 95% Confidence Limits shown as bold and lighter line with vertical bars **(C)**. Decision Curve Analysis for **(A)** nomogram.

**Figure 2A** shows a nomogram resulting from logistic regression fits using Eccentricity (0.45 ACE threshold), SCR after regularization, and SCR after filtering by removing 3 principal components. For a given patient, each component’s contribution is determined by projecting their values onto the “Points.” The total points are computed by summing each of the contributions. “Total points” is projected onto the “Risk of PCa” axis to determine the probability that a given patient suffers from clinically relevant prostate cancer.

**Figure 2B** shows an example of a ROC curve (shown as a bold black line) that displays the Sensitivity plotted against (1-Specificity) (the Specificity value is decreasing along the axis). The bold black line corresponding to the AUC (0.882) and the vertical lines in the ROC curve correspond to the 95% Confidence interval for the AUC. This particular ROC evaluates the logistic fit to Eccentricity, regularized SCR, and SCR filtered by deleting 3 principal components.

Associated with the nomogram is the Decision Curve Analysis (**Figure 2C**). **Figure 2C** shows the net benefit from using each component (eccentricity, regularized SCR, SCR after removing 3 principal components), all components in the regression fit, as a function of Threshold Probability or expected likelihood that the patient has clinically significant prostate cancer. In addition, the net benefit of treating all patient and treating no patients are shown as a standard reference. Applying the regression fit generates the highest net benefit for all threshold probability values relative to applying the individual components (eccentricity, SCR).

**Figure 3:**
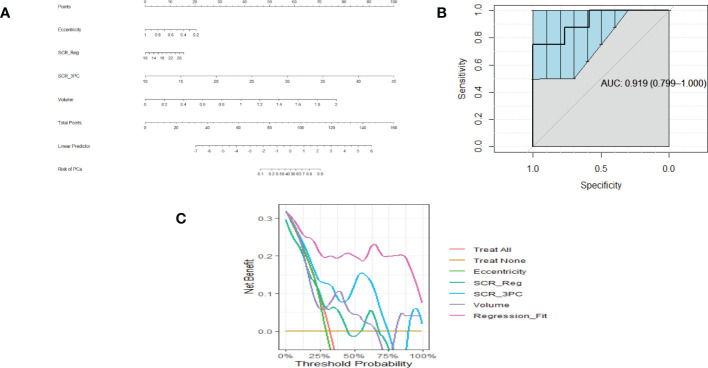
**(A)** is a nomogram resulting from logistic fit to Gleason score with eccentricity, tumor volume, and SCR with 3 PC removed. **(B)** Receiver Operator Curve applied to Logistic Regression for eccentricity, 3 PCs removed from SCR, tumor volume. Area Under the Curve (AUC) and 95% Confidence Limits shown as bold and lighter line with vertical bars. Area Under the Curve (AUC) and 95% Confidence Limits shown as bold and lighter line with vertical bars **(C)**. Decision Curve Analysis for **(A)** nomogram.

Similarly, **Figures 3A–C** show a nomogram, ROC curve, and a Decision Curve Analysis resulting from fitting Eccentricity (0.45 ACE threshold), SCR after regularization, and SCR after filtering by removing 3 principal components, Volume (0.65 ACE threshold). Again, applying the regression fit generates the highest net benefit for all threshold probability values relative to applying the individual components (eccentricity, SCR, volume).

## Discussion

This study is the first to generate a nomogram using features derived from algorithms applied to spatially registered MP-MRI (25–30). Previous studies formed a foundation for the present study, although this study is novel and extended the findings to generate and evaluate the probability for tumor aggressiveness. In addition, the DCA provides an additional tool for guiding application of the nomogram, guiding which input and fits should be employed, and under what conditions. From the high AUC (>0.85), high R2 (>0.70), and low p-values (<0.05), this pilot study found that nomograms can accurately predict the probability of prostate tumor aggressiveness. The nomogram performance as described by AUC from ROC curves is comparable with other studies (18–23) that use metadata such age, clinical data such as PSA, and PI-RADS and that achieve AUC ranging from 0.8 to 0.90.

The transformation of remote sensing-based approaches and algorithms for prostate cancer evaluation discussed in this manuscript forms only a part of the research constellation. There has been considerable progress and research in using biomarkers (47) and multi-parametric MRI (48) to determine the possible presence of prostate cancer and their role in disease management. Companies have translated bench research (47) in biomarkers into clinical tests for their efficacy and offer promising alternatives to the standard prostate serum antigen. Studies investigated the effectiveness of how multi-parametric MRI is employed (49) in the clinic and alternative, simpler configurations and approaches (50) that may eventually make MP-MRI more accommodating for patients and the clinic. Future research may combine the approaches applied to spatially registered hyperspectral hypercubes discussed in this study with biomarkers (47) and may also be modified with the aid of insights gained from MP-MRI implementation (48).

The metrics (AUC, p-value) that assess the accuracy of the nomogram for this study were confined to employing features from spatially registered MP-MRIs. The restricted composition of features nevertheless performed as well or better than studies (18–23) that employed more conventional features such as PSA, age, PI-RADS. Adding extra features from the clinic such as age, PSA etc. to the inputs from spatially registered MP-MRI may further increase the accuracy of the prediction for tumor aggressiveness, as in studies that use PI-RADS data.

Logistic Regression fits the input variables to a binary or a categorical variable, in this case the “Risk of PCa,” which can only be 0 (non-clinically relevant PCa) or 1 (clinically relevant PCa). Earlier multi-variable fitting studies treated the Gleason score as a continuous variable. Better fits for each of the independent variables (p<0.03) were achieved in univariable and multivariable fitting when the Gleason score was treated as continuous. Although the overall fitting (shown in **Tables 1**, **2**) achieves high correlation, assessment of a larger number of samples should improve the univariable fitting using the categorical dependent variable, especially for training/test analysis.

The performance of the multivariable fits diminishes slightly when dividing the patients into training and test sets, as is common in most studies. Due to the limited size of this data set, other combinations of training and testing sets were not feasible. Future analyses using larger patient numbers could reduce confidence intervals and bolster confidence in this study’s findings. Nevertheless, the results described in this manuscript merit further studies that employ larger patient sample sizes that may successfully predict prostate tumor aggressiveness.

There is a question of whether the results are robust or fundamentally unchanged upon using differing target signatures and normal prostate outlines. For a number (but not all) of patients, calculations were rerun with different choice of signatures and different contouring of the normal prostate. The resulting calculations generated virtually the same as those using initial input data. However, a more definitive study is merited.

This study has some limitations. The patients in this study all originated from a single institution (NIH), potentially limiting generalizability. Furthermore, although all patients were prospectively enrolled, this is a retrospective analysis of the data and may be subject to biases. Furthermore, the dataset comprised only 25 patients. Although a small number of patients were assessed, consecutive patients were analyzed to minimize potential bias. Despite this being a pilot analysis with a limited dataset, highly statistically significant P values, high AUC, high coefficient of determination values, and high net benefits in the decision analysis curves were achieved, showing potential clinical value of this approach.

## Conclusions

This retrospective pilot study shows that nomograms that only use metrics from spatially registered MP-MRI achieve comparable performance relative to nomograms that use prostate serum antigen, age, PI-RADS. Validation of these finds from larger and multicenter cohorts are needed before clinical implementation.

## Data availability statement

The raw data supporting the conclusions of this article will be made available by the authors, without undue reservation.

## Ethics statement

Ethical review and approval was not required for the study on human participants in accordance with the local legislation and institutional requirements. The patients/participants provided their written informed consent to participate in this study.

## Author contributions

(I) Conception and design: RM. (II) Administrative support: RM, CS, PC. (III) Provision of study materials or patients: BT, PC. (IV) Collection and assembly of data: RM, BT, PC. (V) Data analysis and interpretation: RM. (VI) Manuscript writing: All authors. (VII) Final approval of manuscript: All authors.

## Appendix

### Overall quantitative metrics description: SCR

The SCR (30–33) is given by

$$SCR={\left(S-\mu \right)}^{T}CM^{-1}(S-\mu )\;(1)$$

that is a matrix multiplication over MP-MRI modalities. The superscript T denotes a vector transpose operation, CM is the covariance matrix, and the superscript -1 denotes a matrix inverse operation, where S is the vector tumor signature or mean over the identified tumor voxels. Vector μ is the mean value for normal prostate or background.

### SCR: Filtering noise

The filtered SCR_Filtered_ is given by

$$SCR_{Filtered}={\left(S-\mu \right)}^{T}CM_{Filtered}^{-1}(S-\mu )\;(2)$$

where the inverse covariance matrix ${\text{CM}}_{\text{Filtered}}^{-1}$ is a square symmetrical matrix and decomposes into three parts (34),

$$CM_{Filtered}^{-1}=\Lambda^{T}\lambda_{Filtered}^{-1}\Lambda \;(3)$$

$$\lambda_{Filtered}^{-1}=\left[\begin{array}{ccccc} \frac{1}{\lambda_{1}^{2}} & 0 & 0 & 0\\ 0 & \frac{1}{\lambda_{2}^{2}} & 0\\ \ldots & \ldots & \ldots \\ 0 & 0 & 0\\ 0 & 0 & \ldots & 0 & 0 \end{array}\right]\;(4)$$

The eigenvalues are ordered according to size ranging from the largest Λ_1_ to the smallest Λ_M_. For unfiltered processing, the images corresponding to the eigenvalues and eigenvectors range from high signal and variation (1, 2) to low variation and very noisy (M-1, M. Filtering out the noisy eigenvectors (29, 31, 35) means removing or deleting the lowest valued eigenvalues (3 or 4 in this study) (Eq 3,4) from the inverse matrix (see Eq 4).

### Regularization and shrinkage

Shrinkage and regularization (29, 36) perturbs the covariance matrix CM(γ) to maximize the normal distribution, or equivalently minimize the discriminant function d(γ) [=-ln(normal distribution)] by adding a diagonal component that is controlled by the parameter γ. This study examines two types of regularization: regularization and modified regularization. Both follow the same procedure but differ in the mixture component. Specifically, the modified regularized SCR_Mod_reg_

SCRmod_Reg(γ=γmin)=(S−μ)TCMmod_Reg−1(γ=γmin)(S−μ)   (5)

where CM_mod___Reg_(γ) is

CMmod_Reg(γ)=(1−γ)CM+γV    (6)

and V is a diagonal matrix filled up with the square of the standard deviations from M modalities and is given by

$$V=\left[\begin{array}{ccccc} \sigma_{1}^{2} & 0 & 0 & 0\\ 0 & \sigma_{2}^{2} & 0\\ \ldots & \ldots & \ldots \\ 0 & \sigma_{M-1}^{2} & 0\\ 0 & 0 & \ldots & 0 & \sigma_{M}^{2} \end{array}\right]\;(7)$$

Using Eqs. [6,7] the modified discriminant function d_mod_Reg_ (γ)

dmod_Reg(γ)=∑i=1N(xi−μ)TCMmod_Reg−1(γ)(xi−μ)+ln(det(CMmod_Reg(γ)))   (8)

is computed for 0<γ<1 and a minimum d_mod_(γ_min_) is found at γ_min_ resulting in a SCR_Mod_Reg_ (Eq. [5]) using a modified regularization procedure (using Eqs. [6,7]).

For SCR_Reg_, the CM_Reg_ uses a matrix containing identical components (proportional to the identity matrix and is simply the average standard deviation σ.

CMReg(γ)=(1−γ)CM+γTra(CM)MI   (9)

by control value γ where Tra denotes the trace operator and I is the identity matrix. γ ranges from γ=0.0 or no CM modification to γ=1.0 or CM is proportional to the identity matrix. Again, the covariance matrix CM_Reg_(γ) is perturbed to maximize the normal distribution, or equivalently minimize the discriminant function d(γ) (=-ln(normal distribution)) by using CM_Reg_ (Eq 9) i.e.

dReg(γ)=∑iN(xi−μ)TCMReg(γ)−1(xi−μ)+ln(det(CMReg(γ)))  (11)

The SC_Reg_ is given by

SCRReg(γ=γmin)=(S−μ)TCMReg−1(γ=γmin)(S−μ)   (9)

### Tumor volume measurements, supervised target detection

The procedure for estimating the tumor volume using the supervised target detection algorithm or ACE has been previously described (26). For spatially-registered MP-MRI, threshold is applied to the ACE map. Voxels exceeding a threshold for ACE scores are assigned to tumor and normal tissue are assigned to ACE scores residing below the threshold. Earlier study (26) examined thresholds 0.40 to 0.85 assessed in 0.05 increments and found that 0.65 was optimal. The number of tumor voxels are converted to volume based on the MRI spatial resolution (1 mm × 1 mm) and slice separation (6 mm) resulting in a voxel volume (r=0.006 cm^3^). Each blob’s volume V_k_ is given by a total number of pixels within each blob and corrected by the voxel volume r (assuming density of unity for each voxel),

$$V_{k}=rN=r\sum_{i=1}^{N}\frac{x_{i}}{abs(x_{i})}\;(12)$$
